# CARving up colorectal cancer organoids in vitro

**DOI:** 10.1038/s41435-019-0079-7

**Published:** 2019-05-20

**Authors:** Christian M. Schürch

**Affiliations:** Baxter Laboratory for Stem Cell Biology, Department of Microbiology and Immunology, Stanford University School of Medicine, Stanford, CA USA

**Keywords:** Immunotherapy, Tumour immunology, Imaging the immune system, Cell death and immune response

Colorectal cancer (CRC) is one of the leading causes of cancer morbidity and mortality worldwide [1]. Over the last decades, the introduction of screening tests, changes in lifestyle and risk factors, and advances in surgery and diagnostics have reduced CRC incidence and mortality. In addition, clinical outcomes of patients with metastatic disease (mCRC) have improved substantially, with median overall survival exceeding 30 months in the era of combination systemic chemotherapy, targeted therapies, and resection or ablation of localized metastases [2–4]. This is reflected by an increasing number of mCRC patients treated within the framework of clinical trials or specialized cancer centers, and is paralleled by the development of novel imaging modalities and molecular tests to monitor these patients. Nevertheless, unfortunately, the majority of mCRC patients are still not definitely cured and will ultimately succumb to their disease.

Chimeric antigen receptor (CAR) T cells have induced spectacular remissions and are now approved for use in patients with relapsed/refractory hematological malignancies. This has revived the interest in using CAR T cells and natural killer (NK) cells in solid tumors such as CRC, especially in the metastatic setting, and drives the need for the identification of antigen targets, and the development of preclinical models to test these therapies [5, 6]. One of the many important key prerequisites for CAR T cell and NK cell therapies to be effective in solid tumors is the ability of the CAR cells to penetrate into, and survive in, the tumor microenvironment (TME). The TME consists of vasculature, stroma, innate and adaptive immune cell subsets, and extracellular matrix (ECM), and is the primary location of tumor cell−immune cell interactions. In many solid tumors, the TME is immunosuppressive by preventing effective recruitment of tumor-specific adaptive immune cells (chemokine mismatch, aberrant vasculature, and ECM barriers) and/or by preventing their survival and effector functions (expression of checkpoint receptor ligands, recruitment of regulatory T cells, myeloid-derived suppressor cells, etc.) [7]. Therefore, in order for CAR cell therapies to be improved and to ultimately succeed also in solid tumors, it is necessary to better understand these cells’ interactions, mobility, survival, and effector functions in the context of the TME.

Writing in the *EMBO* *Journal*, Schnalzger et al. report an elegant in vitro platform for preclinical testing of CAR cells using patient-derived CRC organoids [8]. This is an important step toward the accomplishment of above-mentioned goals and is well in line with ongoing efforts toward personalized cancer medicine. First, the authors modified the organoid culture conditions such that CAR NK cell killing can occur. Nicotinamide (vitamin B3), a supplement present in standard organoid culture media, was identified as an NK cell-inhibitory factor, and removing nicotinamide restored NK cell killing without affecting organoid viability. In addition, the authors found that culturing organoids on top of a matrigel scaffold, as compared to within the matrigel or in suspension, resulted in effective CAR NK cell-mediated organoid killing. The observation that epithelial cell adhesion molecule (EPCAM)-specific CAR NK cells were unable to kill normal colon organoids when these were cultured within the matrigel or in suspension indicates that CAR cells need an ECM scaffold to perform their function, probably for attachment and crawling, while simultaneously being unable to penetrate into this scaffold. This is an important finding that warrants future investigation to dissect the mechanisms of CAR cell−ECM interactions.

Second, the authors optimized their system by retrovirally transducing the organoids with a luciferase-GFP vector to monitor the extent of killing after different time points. On one hand, the introduction of luciferase eliminates the need for microscopic counting of organoids and significantly simplifies the experimental setup, allowing to collect and store lysed supernatants, and increasing the throughput of this platform. On the other hand, GFP expression, in conjunction with CD45 staining of NK cells, allows for live-cell microscopy-based visualization and quantification of killing at the single-organoid level. Importantly, both luciferase- and live-imaging-based experimental readouts yielded similar results.

**Fig. 1:**
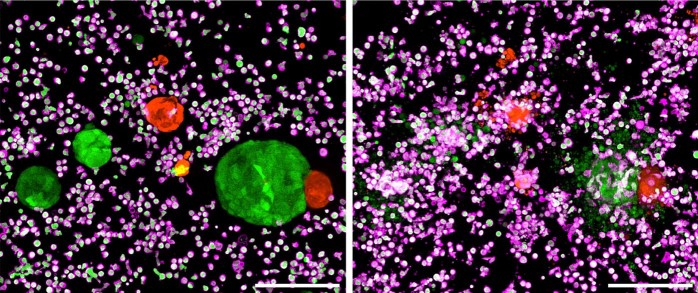
Live microscopy of CRC organoid killing in vitro. EGFRvIII-transduced CRC organoids (GFP-positive, green) were co-cultured with normal colon organoids (DsRED-positive, red) and EGFRvIII-specific CAR NK cells (GFP-positive, green) stained with anti-CD45-APC (magenta). Two different time points are shown (left panel, 0 h; right panel, 10 h). Maximal intensity projection images, scale bars 200 mm. Images courtesy of Henner Farin, Goethe University Frankfurt

The authors then expanded their model to two clinically relevant cancer-associated antigens, epidermal growth factor receptor, variant III (EGFRvIII) and FRIZZLED (FZD), the WNT receptor. EGFRvIII, a constitutively active EGFR mutant lacking exons 2–7, is a neoantigen frequently amplified in solid tumors [9]. FZD is overexpressed in a subset of CRCs that lack RNF43/ZNRF3, a receptor that complexes with LGR-5 and negatively regulates WNT signaling by promoting FZD internalization and degradation in response to R-spondin [10]. EGFRvIII-specific CAR NK cells were highly selective toward organoids lentivirally transduced with this EGFR mutant, and no off-target toxicity was observed in a competitive killing assay in the presence of healthy colon organoids (Fig. 1). In contrast, FZD-specific CAR NK cells killed organoids regardless of their FZD receptor status, indicating that a clinical strategy targeting FZD would likely result in mucosal toxicity.

Although the study by Schnalzger et al. is an important step toward translating the preclinical testing of CAR cells into personalized cancer medicine, several key questions remain open. First, the results with FZD-targeting NK cells that also destroyed normal colon organoids emphasize that optimal antigenic targets for mCRC need to be identified. Recently, several clinical trials for CAR cell therapy of solid tumors such as prostate cancer (target: prostate-specific membrane antigen (PSMA)), mesothelioma, pancreatic and ovarian cancer (target: mesothelin), and glioma (targets: EGFR, EGFRvIII, and HER2) have been initiated [5]. For CRC, preclinical models using CAR T cells directed against guanylyl cyclase C (GUCY2C), an enzyme overexpressed in CRC metastases, showed promising therapeutic activity, and a GUC2YC vaccination was well tolerated in 10 CRC patients without metastases (ClinicalTrials.gov NCT01972737) [11–13]. An advantage of GUC2YC as a CAR target is that its expression is restricted to the apical (luminal) surface in normal epithelia, a feature that is lost in metastases, rendering them visible to the CAR cells. However, GUC2YC is also expressed by a subset of hypothalamic neurons, and neurotoxicity is therefore a caveat [13].

Second, the ideal effector-to-target (E:T) cell ratio has to be determined to allow for optimal tumor cell killing with minimal side effects in vivo. In their in vitro model, the authors observed increasing killing at E:T ratios starting from 0.5:1, with highest efficacy at 4:1. However, how these ratios translate into a clinical setting is still ill defined.

Third, the role of TME and ECM and their effects on CAR cell behavior must be better defined in order to understand and improve future CAR cell therapies for solid tumors. The study by Schnalzger et al. is an important step in this direction and their in vitro organoid killing platform paves the way to answer these questions in the future.
